# Hesperidin and Hesperetin: Epigenetic-Stemness Crosstalk, Antitumor Mechanisms, Preclinical Data and Translation Barriers

**DOI:** 10.3390/biom16071063

**Published:** 2026-07-21

**Authors:** Mengqi Guo, Linxin Shao, Huiqing Yin, Qianrui Kou, Lele Shang, Haixia Guan, Fang Li

**Affiliations:** Yan’an Medical College, Yan’an University, Yan’an 716000, China; mengqiguo@stu.yau.edu.cn (M.G.); 1350770360@stu.yau.edu.cn (L.S.); huiqingyin@stu.yau.edu.cn (H.Y.); 13772556944@163.com (Q.K.); leleshang@stu.yau.edu.cn (L.S.); ghx11261260@stu.yau.edu.cn (H.G.)

**Keywords:** hesperidin, hesperetin, epigenetic regulation, cancer stem cells

## Abstract

Hesperidin is a natural flavonoid derived from citrus plants, which can be hydrolyzed into hesperetin in vivo. Both compounds have anti-inflammatory, antioxidant and antitumor activities. At present, there is a lack of reviews focusing on the epigenetic regulation of cancer stem cells (CSCs) mediated by hesperidin and hesperetin. This review summarizes the molecular crosstalk between hesperidin/hesperetin and CSCs mediated via three major epigenetic pathways, including direct regulatory effects, indirect modulatory actions, and mechanistic relationships proposed based on scientific hypotheses. We elaborate their effects on inhibiting the self-renewal, invasion and metastasis of CSCs as well as reversing chemoresistance, and analyze the crosstalk between epigenetic networks and classical signaling pathways of CSCs. Furthermore, we discuss the core bottlenecks restricting the clinical transformation of these two compounds and introduce improvement strategies such as nanodelivery systems. Current research is still confronted with problems including CSC heterogeneity and the potential off-target toxicity of drugs. In conclusion, hesperidin and hesperetin may serve as potential candidate agents for epigenetic regulation targeting CSCs, which can offer novel theoretical basis for comprehensive tumor therapy.

## 1. Introduction

### 1.1. Research Potential of Natural Compounds in Tumor Therapy

The continuously increasing global incidence and mortality of cancer make it urgent to explore the pathogenesis of cancer and identify new therapeutic targets and drugs [1]. Natural flavonoids have attracted extensive attention in tumor research due to their good biocompatibility and multi-target regulatory effects [2]. Hesperidin, a typical flavonoid widely distributed in citrus plants, exerts anticancer, anti-inflammatory and antioxidant effects. Hesperetin can be generated from hesperidin via hydrolysis catalyzed by intestinal rhamnosidase, and it can also be extracted by industrial methods. This transformation process may serve as an important basis for its antitumor activity [3,4]. Up to now, preliminary antitumor studies of hesperidin and hesperetin have been carried out in various tumor models such as ovarian cancer, breast cancer and leukemia, showing favorable application potential [5,6,7].

### 1.2. Roles of Epigenetic Regulation in Tumor Oncogenesis and Progression

Epigenetic regulation refers to the process of modulating gene expression via DNA methylation, histone modification, non-coding RNA regulation, chromatin remodeling and other approaches without altering DNA sequences [8]. DNA methylation mainly inhibits gene expression by adding methyl groups to CpG islands, and recruits inhibitory methyl-binding proteins to maintain gene silencing [9]. Accumulating studies have demonstrated that aberrant DNA methylation plays a key regulatory role in glioblastoma, gastric cancer and breast cancer [10].

Histone modification refers to post-translational modifications (PTMs) of N-terminal amino acids of histones, including histone methylation, histone acetylation and histone ubiquitination [11]. Histone modification is closely associated with lung cancer, breast cancer, head and neck squamous cell carcinoma and colorectal cancer [12,13,14,15]. Moreover, microRNAs (miRNAs), a type of non-coding RNA, may exert critical functions in the regulation of tumor gene expression [16].

### 1.3. Roles of Cancer Stem Cells in Tumor Oncogenesis and Progression

Cancer stem cells (CSCs) are a cell population with strong self-renewal ability, cellular plasticity and drug resistance, which are key drivers of tumor development and chemoresistance [17]. CSCs can differentiate into multiple tumor cell subtypes, so as to maintain and regulate tumor heterogeneity [18]. Classic CSC biomarkers include CD133, CD44 and ALDH1 [19,20]. CD133 is widely expressed in colorectal cancer, lung cancer, prostate cancer and bladder cancer [21,22,23]. CD44 is commonly detected in liver cancer, breast cancer and head and neck squamous cell carcinoma [24,25,26]. ALDH1 is also recognized as a biomarker of breast cancer. Elevated ALDH1 expression is correlated with HER2 positivity and is often associated with poor prognosis [27]. Besides breast cancer, ALDH1 activity is also widely utilized as a biomarker for CSCs in other solid tumors, including head and neck squamous cell carcinoma, colorectal cancer and lung cancer [28,29,30].

In addition, extensive aberrant DNA methylation exists in CSCs, leading to the silencing of tumor suppressor genes and activation of CSC-related genes [31]. Epigenetic modifications and CSCs may cooperatively modulate tumor growth, metastasis, and chemoresistance [32]. Based on the above background, this review centers on hesperidin and hesperetin, and elaborates their molecular crosstalk with CSCs through epigenetic modifications, alongside their pharmacological properties and obstacles in clinical translation, aiming to lay a foundation for fundamental research and translational development of these natural flavonoids.

## 2. Regulatory Effects of Hesperidin and Hesperetin on Tumor Growth, Metastasis and Chemoresistance

Benefiting from the characteristic of multi-target regulation, hesperidin shows great potential in inhibiting tumor growth and metastasis and alleviating tumor cell chemoresistance. In the group treated with hesperidin (10 μmol/L) combined with paclitaxel (PTX, 10 μmol/L), the cell viability was 16.38%, the proportion of apoptotic cells reached 83.68%, and the number of migratory cells decreased by 65%, which was significantly different from the group treated with PTX (20 μmol/L) alone. The tumor inhibition rate of combined treatment with hesperidin and PTX was 66.17%. Hesperidin may induce apoptosis of oral squamous cell carcinoma (OSCC) cells by upregulating the expression of Bax and caspase-3. Meanwhile, it significantly downregulates MMP-9 and N-cadherin, key molecules involved in EMT, and upregulates E-cadherin, thereby reducing the migration ability of OSCC cells [33].

In vitro combined treatment with hesperetin (200, 400, 600, 800 μM) and 5-fluorouracil (5-FU, 20, 40, 60, 80 μM) as well as in vivo intervention with hesperetin (60 mg/kg) combined with 5-FU (10 mg/kg) achieved better antitumor effects than monotherapy. The combination arrested esophageal cancer cells at the G0/G1 phase, inhibits the PI3K/AKT signaling pathway, upregulates p21, and downregulates Cyclin D1, MMP-2 and MMP-9, thereby hindering the proliferation and invasion of esophageal cancer cells (Eca-109). Hesperetin combined with 5-FU activated the mitochondrial apoptotic pathway, reduced the Bcl-2/Bax ratio, and triggered the activation of caspase-3 and caspase-9 to induce the apoptosis of esophageal cancer cells [34].

In the Ehrlich ascites carcinoma (EAC) mouse model, combined administration of hesperidin (100 mg/kg) and cisplatin (5 mg/kg) exerted synergistic anticancer effects, which were superior to single-drug treatment. The combination further downregulated the tumor proliferation marker Ki-67 and upregulated the apoptotic effector protein caspase-3, enhancing the effects of cisplatin on inhibiting tumor proliferation and inducing cell apoptosis [35]. After treatment with 100 μM and 200 μM hesperidin alone, the cell viability decreased by 45.1% and 65.2% respectively. After treatment with 25 μM and 50 μM cisplatin alone, the cell viability decreased by 37.4% and 48.4% respectively. Compared with cisplatin monotherapy and hesperidin monotherapy, the cell viability of malignant melanoma cells (A431) was reduced to 21.7% ± 2.8% in the group treated with 108.4 μM hesperidin combined with 32.8 μM cisplatin. The two agents synergistically upregulated the expression and protein activity of pro-apoptotic genes Bax, caspase-3 and caspase-7, and significantly downregulated the anti-apoptotic gene Survivin, which greatly increased the apoptosis rate of malignant melanoma cells and enhanced cytotoxicity [36].

Combined treatment with hesperidin (25 μM) and cisplatin (25 μM) significantly enhanced the inhibitory effect on laryngeal carcinoma Hep-2 cells by regulating the TRPM2 channel compared with monotherapy. Activated TRPM2 mediated massive extracellular Ca^2+^ influx and disrupted cellular calcium homeostasis. Meanwhile, it induced abnormal accumulation of ROS, depletion of glutathione (GSH) and elevation of malondialdehyde (MDA), aggravating cellular oxidative stress and lipid peroxidation damage. The mitochondrial membrane potential (ΔΨm) decreased, leading to mitochondrial dysfunction. In addition, the combination of hesperidin and cisplatin upregulated the expression of pro-inflammatory cytokines IL-1β and TNF-α, and ultimately induced apoptosis of human laryngeal squamous cell carcinoma Hep-2 cells, effectively enhancing the in vitro antitumor cytotoxicity of cisplatin [37].

Combined treatment with hesperidin (50–200 μM) and gemcitabine (10–50 μM) exerted synergistic anti-proliferative effects on human endometrial carcinoma cells ISHIKAWA. The most prominent effect was observed in the group treated with hesperidin (100 μM) plus gemcitabine (10 μM), with a tumor inhibition rate of 38–42% at 24 h and 26–30% at 48 h. The related mechanisms are as follows: Firstly, hesperidin combined with gemcitabine induced cell apoptosis by regulating the mitochondrial apoptotic pathway, including upregulating pro-apoptotic protein Bax, downregulating anti-apoptotic protein Bcl-2, and activating caspase-3/7 protease activity. Meanwhile, it downregulated HIF-1α and VEGF expression to block tumor hypoxia adaptation and angiogenesis. In terms of redox homeostasis, hesperidin neutralized excessive reactive oxygen species (ROS) induced by gemcitabine, realizing dual effects of chemotherapy sensitization and toxicity reduction [38].

Compared with monotherapy, combined treatment with hesperidin (10–1000 μM) and doxorubicin (10–1000 nM) significantly increased caspase-3 level; decreased Bcl-2, IL-1β, IL-6 and TNF-α levels; and promoted apoptosis of HeLa cervical cancer cells [39]. Furthermore, compared with sorafenib monotherapy, combined treatment with 1.5 mmol/L hesperidin and 1.0 μmol/L sorafenib remarkably reduced the expression of CSC markers (CD44, CD133) and β-catenin in hepatocellular carcinoma cells, and significantly increased caspase-3 expression, thereby strengthening the inhibitory effect on proliferation and pro-apoptotic effect of HepG2 cells [40].

Collectively, in vitro and in vivo experiments have verified that the combination of hesperidin or hesperetin with platinum drugs, taxanes, fluorouracil, gemcitabine and other chemotherapeutic agents can exert synergistic antitumor effects through multiple mechanisms, including activating the mitochondrial apoptotic pathway, inhibiting EMT, eliminating excessive ROS and blocking angiogenesis. Certain combination therapeutic regimens can also alleviate the oxidative damage induced by chemotherapeutic agents, thereby exerting synergistic antitumor effects (Table 1).

## 3. Key Signaling Pathways and Molecular Targets of Hesperidin and Hesperetin in Regulating Cancer Stem Cells and Epigenetics

### 3.1. Molecular Mechanisms of Hesperidin and Hesperetin in Tumor Epigenetic Modification

Epigenetic modification plays an important role in tumor development and progression, and the regulation of epigenetics by natural drugs has become a hot research topic in recent years [41].

#### 3.1.1. Direct Regulation

Studies have confirmed that treatment of gastric cancer cells (MKN45, HGC27) with hesperetin (≤100 μM) downregulated the expression of histone acetyltransferase CBP, reduced DOT1L acetylation and degradation, and further decreased H3K79 methylation. This process downregulated CDH2, FN1, Twist, MMP2 and MMP9, and upregulated CDH1, ultimately inhibiting the migration and invasion of gastric cancer cells [42].

MicroRNAs (miRNAs) are important post-transcriptional epigenetic regulators in tumor development [43]. MiR-21-5p and miR-155-5p are abnormally highly expressed in breast cancer, which may accelerate malignant progression by inhibiting tumor cell apoptosis and promoting cell cycle progression, and are considered potential epigenetic therapeutic targets for breast cancer [44]. After breast cancer cells (MCF-7) were intervened with hesperidin nanoemulsion (29.36 μg/mL, 100 μg/mL) for 24 h, the transcriptional levels of miR-21 and miR-155 were significantly downregulated, arresting the cell cycle at the G2/M phase and further promoting the apoptosis of the breast cancer cells [45].

Other researchers treated breast cancer cells (MDA-MB-231) with hesperetin (10–500 μM). The results showed that there was a unique interaction between miR-486-5p and lncRNA H19, and the two presented an obvious bidirectional regulatory relationship. Hesperetin may upregulate miR-486-5p to directly downregulate lncRNA H19 and ICAM-1 expression. It can also inhibit the expression of downstream ICAM-1 by downregulating lncRNA H19, and ultimately suppress the proliferation, colony formation and migration of breast cancer cells [46].

#### 3.1.2. Indirect Regulation

Treatment of breast cancer cells (BT-474, SKBR3) with hesperetin (200, 400, 600 μM) significantly upregulated the expression of MLH1 and MSH2. As core genes of the DNA mismatch repair system, the upregulation of MLH1 and MSH2 is obviously associated with the promotion of breast cancer cell apoptosis [47,48]. Studies have shown that tumor cells and cisplatin-resistant cell lines exhibit hypermethylation in the MLH1 promoter region, revealing a close correlation between MLH1 and epigenetics [49,50]. Hesperetin may activate the mismatch repair pathway to induce tumor cell apoptosis via epigenetic approaches, which remains to be verified by further experiments.

**Figure 1:**
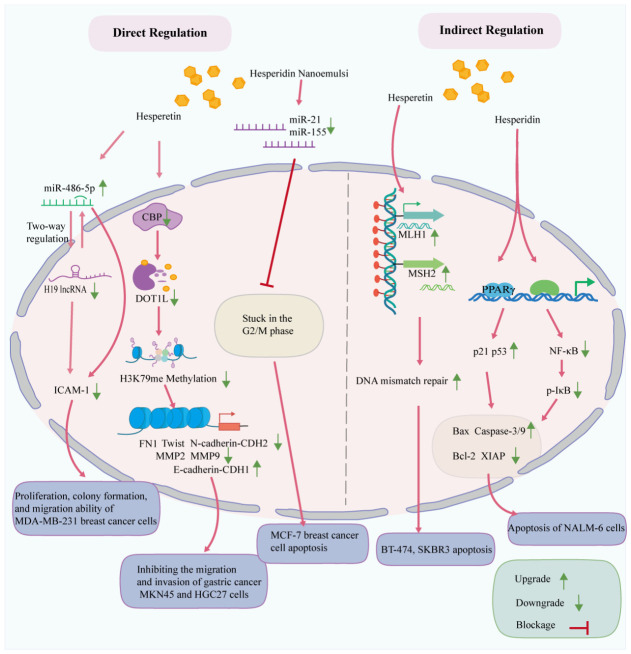
Direct and indirect epigenetic cascades mediating the antitumor activities of hesperetin and hesperidin across gastric and breast cancers and B lymphoblastic leukemia. Their tumor-suppressive functions rely on histone/non-coding RNA epigenetic regulation, as well as modulation of MLH1/MSH2 and PPARγ to induce cell cycle arrest and apoptosis. Green up-arrows: upregulated molecules; green down-arrows: downregulated molecules; red bars: molecular inhibition.

Treatment of human B lymphoblastic leukemia cells (NALM-6) with hesperidin (10–100 μM) for 24 h upregulated PPARγ. On the one hand, it increased the expression of p53 and p21 to induce cell cycle arrest and initiate apoptosis. On the other hand, it inhibited IκB phosphorylation and suppressed NF-κB activity to promote tumor cell apoptosis. Meanwhile, hesperidin upregulated Bax, downregulated Bcl-2 and XIAP, and activated caspase-3/9 within 24 h, jointly inducing apoptosis of NALM-6 cells [51]. It has been reported that PPARγ is a target of epigenetic modification and plays an important role in tumor regulation [52]. Therefore, hesperidin may exert antitumor effects via epigenetically regulating PPARγ (Figure 1).

### 3.2. Molecular Mechanisms of Hesperidin and Hesperetin in Regulating CSCs

CSC-related transcription factors including OCT4, SOX2, NANOG, MYC, CCND1, LGR5 and CXCR-4 integrate multiple signaling pathways such as Wnt/β-catenin, Notch, NF-κB, TGF-β/SMAD and JAK-STAT to form a molecular regulatory network, thereby regulating CSC stemness [53,54,55,56].

#### 3.2.1. Direct Regulation

Firstly, hesperidin may regulate transcription factors and signaling pathways to inhibit CSCs. Treatment of breast cancer cells (MDA-MB-231) with hesperidin (12.5–100 μM) stabilized the G-quadruplex silencing element Pu-27 in the c-Myc promoter region, inhibited c-Myc transcription and downregulated the expression of downstream CCND1, which may block the proliferation and impair the self-renewal ability of breast CSCs [57].

Treatment of non-small cell lung cancer cells (A549, H460, H1975) with hesperidin (25–62.5 μg/mL) inhibited SDF-1α secretion and downregulated CXCR-4 to block the activation of the SDF-1/CXCR-4 signaling axis. This process reduced the expression of NF-κB (p-p65, p-IκB) and PI3K/Akt (p-Akt), further downregulated Vimentin and MMP-9, upregulated CK-19, reversed EMT, and inhibited the migration and invasion of lung cancer cells [58].

Studies have demonstrated that the self-renewal, proliferation and drug resistance of CSCs are mainly regulated by the classical Wnt and Notch signaling pathways [59,60]. In vitro treatment with 0.5 mmol/L and 1.5 mmol/L hesperidin and in vivo administration at 400 mg/kg blocked the Wnt pathway and inhibited the nuclear translocation of β-catenin, thereby downregulating CD44 and CD133. Meanwhile, it upregulated Bax and caspase-3 and downregulated Bcl-2, ultimately inducing apoptosis of hepatocellular carcinoma cells [40].

Treatment of breast cancer MCF-7 cells with hesperidin (100 μM, 200 μM) inhibited the PI3K/Akt pathway, downregulated MDM2 and upregulated p53 expression. Further regulation by p53 includes inducing G0/G1 cell cycle arrest to inhibit cell proliferation, activating apoptotic signals to promote cell death, downregulating ALDH1 to suppress breast CSC stemness, spheroid formation and colony formation, and downregulating MMP9 to inhibit cell migration and invasion. Combined with bioinformatics analysis, p53 was identified as the core target of hesperidin acting on breast CSCs, and the targeted regulatory mechanism between them needs further in-depth research [61].

#### 3.2.2. Indirect Regulation

**Figure 2:**
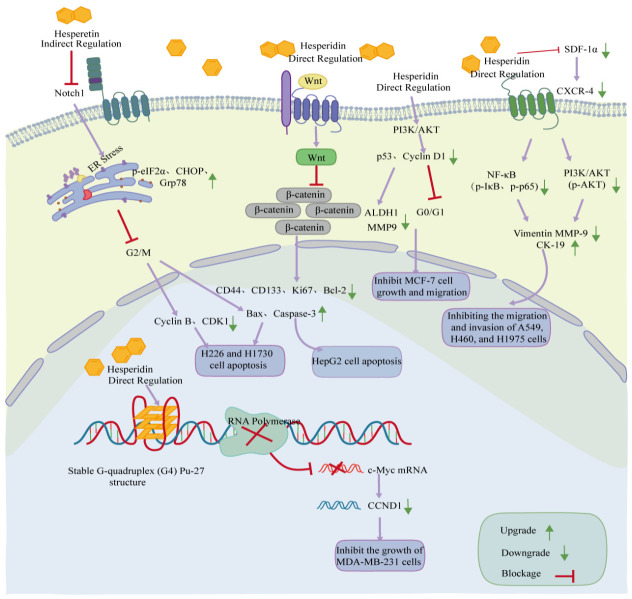
Putative multi-pathway networks underlying the anti-stemness, anti-proliferative and anti-metastatic activities of hesperetin and hesperidin in breast, lung and liver cancer models. Hesperetin triggers G2/M arrest and lung cancer apoptosis via Notch1/ER stress. Hesperidin suppresses CSC phenotypes, cell cycle progression and EMT through Wnt/β-catenin, PI3K/AKT, CXCR4/NF-κB and c-Myc G-quadruplex signaling. Green up-arrows: upregulated molecules; green down-arrows: downregulated molecules; red bars: molecular inhibition.

Treatment of lung squamous cell carcinoma cells with hesperetin (H1703: 37.5–150 μM, H226: 75–300 μM) inhibited the Notch1 signaling pathway and activated endoplasmic reticulum stress-related proteins p-eIf2α, CHOP and Grp78. After the treatment of hesperetin, tumor cells were arrested at the G2/M phase, accompanied by the upregulation of pro-apoptotic protein Bax and Cleaved-Caspase-3, downregulation of Cyclin B and CKD1, and decreased mitochondrial membrane potential, which ultimately induced apoptosis of lung squamous cell carcinoma cells [62]. Given that Notch1 can promote the nuclear translocation of β-catenin, the dual inhibition of Wnt/β-catenin and Notch pathways by hesperidin blocks the crosstalk between the two signaling pathways, which may serve as one of the potential mechanisms underlying its suppressive activity against CSCs [63] (Figure 2).

### 3.3. Crosstalk Between Epigenetic Modification and CSCs: Mechanisms Underlying the Effects of Hesperidin and Hesperetin

Extensive aberrant DNA methylation exists in CSCs, and the inhibition of DNA methyltransferases (DNMTs) can reduce the CSC pool and suppress tumorigenesis [64,65]. Histone acetylation and methylation can remodel chromatin structure and affect the transcriptional activity of CSC-related genes [66]. In addition, epigenetic regulatory mechanisms such as DNA methylation, non-coding RNA and histone modification may interact with CSC-related signaling pathways including Wnt/β-catenin, Notch and Hedgehog [67,68,69].

#### 3.3.1. Direct Regulation

Treatment of non-small cell lung cancer cells (A549, H460) and xenograft tumors with hesperidin (1 μM, 2.5 μM in vitro; 60 mg/kg in vivo) upregulated miR-132, which directly targeted and silenced the CSC-related gene ZEB2, thereby exerting anti-proliferative and pro-apoptotic effects [70,71]. The above findings provide references for the study on the regulation of CSCs by hesperidin via epigenetics.

#### 3.3.2. Indirect Regulation

Studies have shown that treatment of hepatocellular carcinoma cells (HepG2) in vitro and intervention in rat liver cancer models in vivo with hesperidin (50–400 μM in vitro; 150 mg/kg in vivo) inhibited both canonical Wnt3a/β-catenin and non-canonical Wnt5a pathways, downregulated β-catenin and its downstream Cyclin D1, upregulated Caspase-3, and induced the apoptosis of hepatocellular carcinoma cells [72]. Hypermethylation of the Wnt5a promoter region has been reported during tumor development, and Wnt5a plays an essential role in sustaining the survival and stemness of CSCs [59,73]. However, it remains to be elucidated whether hesperidin regulates CSCs via epigenetic modification of Wnt5a, which requires further comprehensive experimental verification.

#### 3.3.3. Putative Mechanistic Hypothesis

Hesperetin (10–100 μg/mL) binds to TOP2A protein and significantly downregulates TOP2A expression in lung cancer cells (A549), thereby blocking DNA replication and cell proliferation, accompanied by mitochondrial dysfunction and eventual cell apoptosis [74]. TOP2A has been reported to interact with epigenetic regulators such as BAZ2A, KDM1A and EZH2, and to be modulated by RNA epigenetic modifications, particularly m6A methylation [75,76,77]. In non-tumor models, hesperidin (0.25 μM) was shown to downregulate m6A expression in human villous trophoblasts under inflammatory/high-glucose conditions [78]; however, whether hesperidin exerts similar effects on m6A in cancer cells remains unknown. Regarding epigenetic regulation of TOP2A, norcantharidin has been demonstrated to inhibit H3K27me3, which may in turn suppress TOP2A expression and upregulate p53, leading to apoptosis in hepatocellular carcinoma cells [79]. This suggests that epigenetic modifications can regulate TOP2A, but no study has yet reported that hesperidin or hesperetin can regulate TOP2A through epigenetic mechanisms. In prostate cancer, TOP2A negativity is considered a feature of CSCs and is associated with drug resistance [80]. These observations indicate that TOP2A may serve as a potential link between epigenetic modifications and CSC characteristics. Given that hesperidin can modulate both TOP2A expression and m6A levels (in different contexts), it is plausible that hesperidin could influence the epigenetic-TOP2A-CSCs axis, although direct experimental evidence in tumor models is currently lacking [81].

**Figure 3:**
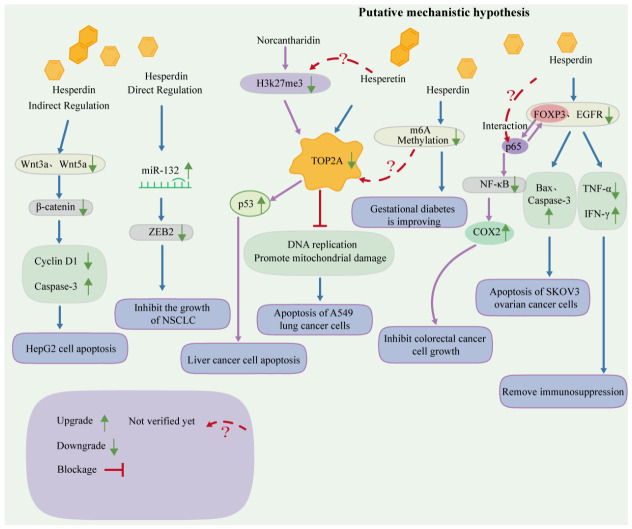
Schematic overview of confirmed and putative epigenetic-CSC regulatory networks modulated by hesperetin and hesperidin. Hesperidin directly suppresses Wnt/β-catenin and miR-132/ZEB2 cascades to induce tumor cell apoptosis and restrain CSC properties. Multiple unvalidated epigenetic axes centered on TOP2A and FOXP3 are hypothesized to bridge hesperetin/hesperidin activity with CSC homeostasis, as marked by red question marks. Green arrows denote altered molecular expression; red bars indicate molecular inhibition.

Hesperidin (10–400 μM) downregulated FOXP3 expression in ovarian cancer cells (SKOV3) via two parallel pathways: Pathway 1: hesperidin upregulated Bax and Caspase-3 to activate the endogenous apoptotic pathway and directly induce apoptosis of SKOV3 cells. Pathway 2: hesperidin downregulated FOXP3 and TNF-α while upregulating IFN-γ, relieving immunosuppression in the tumor microenvironment and remodeling antitumor immunity [82]. Studies have indicated that the induction and maintenance of FOXP3 are accompanied by epigenetic modifications such as histone acetylation and DNA demethylation [83]. In addition, FOXP3 can bind to p65 and inhibit the transcription of the stemness factor COX2 mediated by NF-κB, thereby suppressing CSCs and the malignant progression of colorectal cancer [84]. Therefore, the expression of FOXP3 may be regulated by epigenetic modifications including DNA methylation and histone acetylation; meanwhile, FOXP3 is capable of modulating stemness-related pathways in CSCs. However, whether the regulation of FOXP3 by hesperidin mediates the crosstalk between epigenetics and CSCs remains to be verified by experiments (Figure 3).

## 4. Preclinical Research on Hesperidin: Challenges and Optimization Strategies

### 4.1. Optimization of Drug Delivery Systems: Addressing the Bottleneck of Low Bioavailability

The main obstacle restricting the clinical transformation of hesperidin is its low lipophilicity, which leads to insufficient bioavailability and impairs its absorption and distribution in vivo [85]. Studies have shown that after healthy volunteers consumed orange juice, hesperidin was hydrolyzed into hesperetin in the intestinal tract. The peak plasma concentration of hesperetin was extremely low, only 0.46 μM and 1.28 μM after intake of 0.5 L and 1 L orange juice respectively. Concentrations commonly applied in current in vitro antitumor experiments fall within the range (50 μM–200 μM), yet direct oral administration of hesperidin or hesperetin monomers to human subjects has not been conducted in relevant studies [86]. Thus, extensive clinical trials are still needed to characterize the peak plasma concentrations of orally administered hesperidin and hesperetin monomers in humans.

Hesperidin methyl chalcone (HMC) was synthesized via methylation to alleviate acute renal injury in rats. When administered via intraperitoneal injection, HMC exerted dose-dependent protective effects within the dose range of 0.03–3 mg/kg, and 3 mg/kg was the optimal effective dose. It significantly activated the Nrf2 pathway, inhibited oxidative stress and inflammation, and reversed renal function damage. HMC remarkably improved the lipophilicity and bioavailability of hesperidin, but its antitumor activity has not been verified so far, and experiments are still needed for further exploration [87].

In prostate cancer research, hesperidin and berberine were co-encapsulated into poly(lactic-co-glycolic acid) (PLGA) nanoparticles. The average particle size of PLGA nanoparticles was less than 200 nm with a polydispersity index lower than 0.1. The hesperidin-loaded PLGA nanoparticles presented a spherical morphology with uniform particle size, a hydrodynamic diameter of 76.2 nm and an encapsulation efficiency of 90%. The cumulative drug release rate reached approximately 93% within 144 h. Cell experiments confirmed that such PLGA nanoparticles significantly improved the bioavailability and antitumor activity of hesperidin and reduced the viability of colorectal cancer HCT116 cells, with the optimal effect observed at the concentration of 10 μg/mL [88]. Nevertheless, all relevant experiments were limited to cellular models and lacked in vivo animal verification. Further extensive animal studies are therefore warranted to clarify whether PLGA-encapsulated hesperidin could ameliorate its bioavailability, safety, tumor-targeting performance, and other critical properties in vivo.

Hesperidin was encapsulated into nanoemulsions (HP-NEM) by spontaneous emulsification to improve solubility and enhance the therapeutic effect on breast cancer. Physicochemical characterization showed that the optimized HP-NEM was spherical with a particle size of 305 ± 40.8 nm, a polydispersity index of 0.308 ± 0.04 and an encapsulation efficiency of 93 ± 0.45%. Within 48 h, the cumulative drug release of HP-NEM reached 98.57 ± 0.39% (*w*/*w*), whereas free hesperidin only exhibited a cumulative release of 46.35 ± 0.61% (*w*/*w*). In vitro experiments verified that HP-NEM exerted selective cytotoxicity on breast cancer MCF-7 cells and had no obvious toxicity to normal HEK293 cells [45]. Hesperidin nanoemulsions have also shown favorable anticancer effects in prostate cancer research [89]. Nevertheless, extensive in vivo studies are still warranted to systematically evaluate the bioavailability, biosafety and targeting capability of HP-NEM.

A comprehensive comparison of the three modification strategies is as follows: hesperidin methyl chalcone improves lipophilicity, but only its renoprotective effect has been verified and its antitumor activity remains unknown; PLGA nanoparticles have high encapsulation efficiency and excellent sustained release performance, but lack satisfactory active targeting ability; hesperidin nanoemulsions significantly enhance solubility and exert selective toxicity to tumor cells, while their in vivo stability needs to be optimized. Currently, all delivery systems are still in the stage of cellular and animal experiments and have not been applied in clinical research.

### 4.2. Spatiotemporal Specificity Regulation: Avoiding Damage to Normal Stem Cells

While killing tumor cells, the multi-target characteristics of hesperidin may interfere with the function of normal stem cells. pH-responsive nanocarriers can realize targeted drug release in the tumor microenvironment and reduce adverse effects on normal tissues and stem cells, which is a feasible direction to achieve spatiotemporal-specific regulation [90,91]. However, relevant research reports are limited, and the biosafety and in vivo targeting efficiency of carriers need verification. Therefore, the establishment of tissue-specific delivery methods or conditionally activated prodrugs can improve the accuracy of spatiotemporal specificity during hesperidin intervention [92].

### 4.3. Management of CSC Heterogeneity: Improving Therapeutic Consistency

The prominent heterogeneity of CSCs is a major obstacle for the application of hesperidin. Studies have shown that although hesperidin can inhibit the proliferation of prostate cancer cells, its pharmacological efficacy varies greatly among different tumor cell subpopulations [89]. At present, single-cell technology has been applied to clarify the heterogeneity of primary neuroblastoma, leukemia and CSCs in the tumor microenvironment [93,94]. In addition, tumor organoid technology is used to recapitulate the heterogeneity of hepatocellular carcinoma, gastric cancer and other malignant tumors [95,96]. Although these technologies have not been applied in research on hesperidin and hesperetin, they provide references for solving the problem of CSC heterogeneity in the future.

### 4.4. Safety Evaluation and Dose Optimization: Defining the Therapeutic Window

Preclinical studies have demonstrated that hesperidin exerts prominent antitumor effects at effective dosages. However, the long-term effects of long-term administration on normal stem cells and the synergistic toxicity of combined medication still need further evaluation [97,98]. The PharmaFormer artificial intelligence drug prediction model established by Zhou Yuru and colleagues integrates a large number of preclinical models via transfer learning and constructs an innovative framework for predicting clinical drug responses. Pharmacokinetic–pharmacodynamic models can be used to optimize the dosage [99]. Although this model has not been applied in the research of hesperidin and hesperetin, it provides a direction for establishing a dosage optimization model for hesperidin based on the PharmaFormer framework.

## 5. Conclusions and Future Perspectives

This review summarizes the antitumor mechanisms of hesperidin and its in vivo metabolite hesperetin. This provides a theoretical basis for the hypothesis that hesperidin and hesperetin may interfere with core signaling pathways of CSCs via epigenetic modifications, thereby suppressing the self-renewal, invasion and metastasis of cancer stem cells and reversing chemoresistance in tumor cells. Meanwhile, it offers a novel perspective for future investigations into precise molecular mechanisms underlying hesperidin and hesperetin in the regulation of tumor progression. Multiple in vitro and in vivo experiments have confirmed that the combination of these two compounds with conventional chemotherapeutic drugs exerts prominent synergistic antitumor effects with low intrinsic toxicity.

At present, many problems remain to be solved in this research field. Firstly, the molecular network of crosstalk between epigenetic regulation and CSCs modulated by hesperidin and hesperetin has not been fully elucidated, and potential new targets such as TOP2A, m6A and FOXP3 need further verification. Secondly, poor lipophilicity and low oral bioavailability restrict the in vivo efficacy of the compounds. Thirdly, CSC heterogeneity and the potential impacts of drugs on normal stem cells have not been fully evaluated.

Future research can be carried out from three aspects: (1) combining single-cell epigenomics and tumor organoid models to analyze the specific mechanisms of drug action in different tumors; (2) optimizing pharmaceutical technologies such as nanodelivery systems and responsive prodrugs to improve drug targeting ability and bioavailability; and (3) conducting long-term toxicity assessment and safety evaluation of combined medication, optimizing administration dosage combined with artificial intelligence models, and promoting the clinical transformation of these natural flavonoids.

## Figures and Tables

**Table 1:** Synergistic antitumor effects and molecular mechanisms of hesperidin/hesperetin combined with chemotherapeutics in diverse tumors.

Tumor Type	Combined Chemotherapeutic Drug	Core Biological Effects	Key Molecular Regulatory Mechanisms
Oral squamous cell carcinoma (OSCC)	Paclitaxel (PTX)	Reduce tumor cell viability, increase apoptotic cell proportion, suppress cell migration; achieve high tumor inhibition rate superior to PTX monotherapy	Upregulate Bax and Caspase-3 to trigger apoptosis; downregulate EMT-related MMP-9 and N-cadherin, upregulate E-cadherin to inhibit EMT and cell migration [33]
Esophageal carcinoma (Eca-109 cells)	5-Fluorouracil (5-FU)	Better anti-proliferative and anti-invasive effects than single drug; induce tumor cell apoptosis	1. Arrest cells at G_0_/G_1_ phase, inhibit PI3K/AKT pathway, upregulate p21, downregulate Cyclin D1, MMP-2 and MMP-9; 2. activate mitochondrial apoptotic pathway, reduce Bcl-2/Bax ratio, activate Caspase-3/9 [34]
Malignant melanoma (A431 cells); Ehrlich ascites carcinoma (EAC) mouse model	Cisplatin	Synergistically reduce cell viability; enhance proliferation inhibition and apoptosis induction compared with monotherapy	1. In EAC model: downregulate proliferation marker Ki-67, upregulate Caspase-3; 2. in A431 cells: upregulate pro-apoptotic Bax, Caspase-3/7, downregulate anti-apoptotic Survivin [35,36]
Laryngeal squamous cell carcinoma (Hep-2 cells)	Cisplatin	Strengthen cisplatin-mediated cytotoxicity; aggravate oxidative damage and induce massive tumor cell apoptosis	Activate TRPM2 channel to trigger extracellular Ca^2+^ overload and calcium homeostasis disorder; induce ROS accumulation, GSH depletion and MDA elevation to exacerbate oxidative stress and lipid peroxidation; decrease mitochondrial membrane potential to cause mitochondrial dysfunction; upregulate pro-inflammatory IL-1β and TNF-α [37]
Human endometrial adenocarcinoma (ISHIKAWA cells)	Gemcitabine	Synergistically inhibit tumor proliferation; exert dual effects of chemosensitization and reduce chemotherapy-induced oxidative toxicity	1. Modulate mitochondrial apoptosis: upregulate Bax, downregulate Bcl-2, activate Caspase-3/7; 2. downregulate HIF-1α and VEGF to block tumor angiogenesis and hypoxia adaptation; 3. scavenge excessive ROS to relieve oxidative injury induced by gemcitabine [38]
Cervical cancer (HeLa cells)	Doxorubicin	Promote apoptosis of cervical cancer cells; reduce inflammatory response	Upregulate Caspase-3; downregulate Bcl-2, pro-inflammatory cytokines IL-1β, IL-6 and TNF-α [39]
Hepatocellular carcinoma (HepG2 cells)	Sorafenib	Improve chemosensitivity; inhibit cancer stem cell stemness; suppress cell proliferation and induce apoptosis	Downregulate CSC markers CD44, CD133 and β-catenin; upregulate Caspase-3 [40]

## Data Availability

No new data were created or analyzed in this study. Data sharing is not applicable to this article.

## Abbreviations

ALDH1	Aldehyde dehydrogenase 1
BCL-2	B-cell lymphoma 2
CD	Cluster of differentiation
CDH1	Cadherin 1 (E-cadherin)
CDH2	Cadherin 2 (N-cadherin)
CK-19	Cytokeratin 19
CSCs	Cancer stem cells
CXCR-4	C-X-C chemokine receptor type 4
DNMTs	DNA methyltransferases
DOT1L	DOT1-like histone lysine methyltransferase
EAC	Ehrlich ascites carcinoma
EMT	Epithelial–mesenchymal transition
FN1	Fibronectin 1
FOXP3	Forkhead box P3
GSH	Glutathione
HDACs	Histone deacetylases
HIF-1α	Hypoxia-inducible factor-1α
HMC	Hesperidin methyl chalcone
HP-NEM	Hesperidin nanoemulsions
ICAM-1	Intercellular adhesion molecule 1
IFN-γ	Interferon-gamma
IL-1β	Interleukin-1 beta
IL-6	Interleukin-6
IκB	Inhibitor of nuclear factor kappa-B
JAK-STAT	Janus kinase-signal transducer and activator of transcription
Ki-67	Marker of proliferation Ki-67
lncRNA	Long non-coding RNA
LGR5	Leucine-rich repeat-containing G-protein coupled receptor 5
MDA	Malondialdehyde
MDM2	Mouse double minute 2 homolog
miRNA/miR	MicroRNA
MLH1	MutL homolog 1
MMP	Matrix metalloproteinase
MSH2	MutS homolog 2
MYC/c-Myc	MYC proto-oncogene
NANOG	Nanog homeobox
NF-κB	Nuclear factor kappa-B
Notch	Notch signaling pathway
Nrf2	Nuclear factor erythroid 2-related factor 2
OCT4	Octamer-binding transcription factor 4
OSCC	Oral squamous cell carcinoma
p21	Cyclin-dependent kinase inhibitor 1A
p53	Tumor protein p53
PI3K/AKT	Phosphatidylinositol 3-kinase/Protein kinase B
PLGA	Poly(lactic-co-glycolic acid)
PPARγ	Peroxisome proliferator-activated receptor gamma
PTX	Paclitaxel
ROS	Reactive oxygen species
SDF-1α	Stromal cell-derived factor-1 alpha
SOX2	SRY-box transcription factor 2
Survivin	Baculoviral IAP repeat-containing protein 5
TGF-β/SMAD	Transforming growth factor-beta/Smad signaling pathway
TNF-α	Tumor necrosis factor-alpha
TOP2A	DNA topoisomerase II alpha
TRPM2	Transient receptor potential melastatin 2
VEGF	Vascular endothelial growth factor
Vimentin	Vimentin cytoskeletal protein
Wnt/β-catenin	Wnt/β-catenin signaling pathway
XIAP	X-linked inhibitor of apoptosis protein
ZEB2	Zinc finger E-box binding homeobox 2
ΔΨm	Mitochondrial membrane potential
m6A	N6-methyladenosine
CpG	Cytosine-phosphate-Guanine
5-FU	5-fluorouracil

## References

[1] Bray F., Laversanne M., Sung H., Ferlay J., Siegel R.L., Soerjomataram I., Jemal A. (2024). Global cancer statistics 2022: GLOBOCAN estimates of incidence and mortality worldwide for 36 cancers in 185 countries. CA Cancer J. Clin..

[2] Bendrihem K.A., Mouane A., Azzi M., Mihoubi M.A., Atanassova M., Sawicka B., Zahnit W., Messaoudi M. (2025). The Role of Medicinal Plants in Modulating Epigenetic Mechanisms: Implications for Cancer Prevention and Therapy. Phytother. Res..

[3] Pyrzynska K. (2022). Hesperidin: A Review on Extraction Methods, Stability and Biological Activities. Nutrients.

[4] Choi S.S., Lee S.H., Lee K.A. (2022). A Comparative Study of Hesperetin, Hesperidin and Hesperidin Glucoside: Antioxidant, Anti-Inflammatory, and Antibacterial Activities In Vitro. Antioxidants.

[5] Kharwade R., Mahajan N.M., Telange D.R., Yadav P.N., More S.R. (2026). Folate receptor-targeted PEGylated PLGA nanoparticles for the site-specific delivery of hesperidin in epithelial ovarian cancer. Artif. Cells Nanomed. Biotechnol..

[6] Hu H., Zhang C., Liu Z. (2026). Hesperidin Inhibits Proliferation and Metastasis of Human Breast Cancer Cells by Suppressing MMP-2/9 Activity and Reversing Epithelial-Mesenchymal Transition. Cancer Inform..

[7] Hosseini S.S., Esmailzadeh E., Zangooei M., Bagheri V. (2025). Hesperetin increases membrane progesterone receptor expression in human myeloid leukemia cells and reduces ROS. Med. Oncol..

[8] Jaenisch R., Bird A. (2003). Epigenetic regulation of gene expression: How the genome integrates intrinsic and environmental signals. Nat. Genet..

[9] Moore L.D., Le T., Fan G. (2013). DNA methylation and its basic function. Neuropsychopharmacology.

[10] Jones P.A., Baylin S.B. (2007). The Epigenomics of Cancer. Cell.

[11] Yao W., Hu X., Wang X. (2024). Crossing epigenetic frontiers: The intersection of novel histone modifications and diseases. Signal Transduct. Target. Ther..

[12] Chen G., Zhu X., Li J., Zhang Y., Wang X., Zhang R., Qin X., Chen X., Wang J., Liao W. (2022). Celastrol inhibits lung cancer growth by triggering histone acetylation and acting synergically with HDAC inhibitors. Pharmacol. Res..

[13] Yeruva S.L.H., Zhao F., Miller K.D., Tevaarwerk A.J., Wagner L.I., Gray R.J., Sparano J.A., Connolly R.M. (2018). E2112: Randomized phase iii trial of endocrine therapy plus entinostat/placebo in patients with hormone receptor-positive advanced breast cancer. npj Breast Cancer.

[14] Li M., Ni Y., Wu J., Zou X., Chen Y., Qiu J., Li Y., Cai H., Wang L., Wang F. (2026). NEK8 kinase-mediated lactate increase impairs antitumor immunity decreasing radiotherapy sensitivity in colorectal cancer. Nat. Commun..

[15] Giudice F.S., Pinto D.S., Nör J.E., Squarize C.H., Castilho R.M. (2013). Inhibition of histone deacetylase impacts cancer stem cells and induces epithelial-mesenchyme transition of head and neck cancer. PLoS ONE.

[16] Slack F.J., Chinnaiyan A.M. (2019). The Role of Non-coding RNAs in Oncology. Cell.

[17] Batlle E., Clevers H. (2017). Cancer stem cells revisited. Nat. Med..

[18] Nassar D., Blanpain C. (2016). Cancer Stem Cells: Basic Concepts and Therapeutic Implications. Annu. Rev. Pathol..

[19] Marin J.J.G., Asensio M., Álvarez-Fernández L., Hortelano-Hernandez N., Delgado-Calvo K., Marijuan R.P., Perez-Silva L., Benizri L.O., Gallai D., Lozano E. (2026). Impact of CD44 alternative splicing on the response to anticancer drugs. Biochem. Pharmacol..

[20] Xue Y., Zheng Y., Zhong Y., Li J., Yang T., Hong S., Wang S., Xie J., Xiong W., Yang X. (2026). Modified Fuzheng Yiliu Decoction chemosensitizes colorectal cancer by inhibiting cancer stem cell metabolism and stemness through CAV1/ZNF460/GRP78 signaling. Phytomedicine.

[21] Kholodenko I.V., Saidova A.A., Potashnikova D.M., Arzumanian V.A., Romashin D.D., Tvorogova A.V., Poverennaya E.V., Yarygin K.N., Kim Y.S. (2025). Contrasting Impacts of Targeted Disruption of the Cancer Stem Cell Marker CD133 and Its Epigenetic Regulator TRIM28 in Colorectal Cancer Cells. Int. J. Mol. Sci..

[22] García-Fernández J., Díez-Villares S., Cascallar M., Rivadulla Costa L., Martins A.S., Groba de Antas S., Tarasco M.C., Pantano E., Taiè G., Bertolini G. (2025). Targeted delivery of therapeutics to metastatic lung cancer cells using aptamer-conjugated nanoemulsions. J. Control. Release.

[23] Klose K., Packeiser E.M., Müller P., Granados-Soler J.L., Schille J.T., Goericke-Pesch S., Kietzmann M., Murua Escobar H., Nolte I. (2021). Metformin and sodium dichloroacetate effects on proliferation, apoptosis, and metabolic activity tested alone and in combination in a canine prostate and a bladder cancer cell line. PLoS ONE.

[24] Maehara O., Sato F., Natsuizaka M., Asano A., Kubota Y., Itoh J., Tsunematsu S., Terashita K., Tsukuda Y., Nakai M. (2015). A pivotal role of Krüppel-like factor 5 in regulation of cancer stem-like cells in hepatocellular carcinoma. Cancer Biol. Ther..

[25] Zheng W., Peng W., Qian F., Zhang M., Duan B., Fan Z., Xie Y., Fu X. (2024). Vitamin D suppresses CD133+/CD44 + cancer stem cell stemness by inhibiting NF-κB signaling and reducing NLRP3 expression in triple-negative breast cancer. Cancer Chemother. Pharmacol..

[26] Khammanivong A., Gopalakrishnan R., Dickerson E.B. (2014). SMURF1 silencing diminishes a CD44-high cancer stem cell-like population in head and neck squamous cell carcinoma. Mol. Cancer.

[27] Ginestier C., Hur M.H., Charafe-Jauffret E., Monville F., Dutcher J., Brown M., Jacquemier J., Viens P., Kleer C.G., Liu S. (2007). ALDH1 is a marker of normal and malignant human mammary stem cells and a predictor of poor clinical outcome. Cell Stem Cell.

[28] Bhaijee F., Pepper D.J., Pitman K.T., Bell D. (2012). Cancer stem cells in head and neck squamous cell carcinoma: A review of current knowledge and future applications. Head. Neck.

[29] Huang E.H., Hynes M.J., Zhang T., Ginestier C., Dontu G., Appelman H., Fields J.Z., Wicha M.S., Boman B.M. (2009). Aldehyde dehydrogenase 1 is a marker for normal and malignant human colonic stem cells (SC) and tracks SC overpopulation during colon tumorigenesis. Cancer Res..

[30] Okudela K., Woo T., Mitsui H., Tajiri M., Masuda M., Ohashi K. (2012). Expression of the potential cancer stem cell markers, CD133, CD44, ALDH1, and β-catenin, in primary lung adenocarcinoma—Their prognostic significance. Pathol. Int..

[31] Verona F., Pantina V.D., Modica C., Lo Iacono M., D’Accardo C., Porcelli G., Cricchio D., Turdo A., Gaggianesi M., Di Franco S. (2022). Targeting epigenetic alterations in cancer stem cells. Front. Mol. Med..

[32] Dakal T.C., Bhushan R., Xu C., Gadi B.R., Cameotra S.S., Yadav V., Maciaczyk J., Schmidt-Wolf I.G.H., Kumar A., Sharma A. (2024). Intricate relationship between cancer stemness, metastasis, and drug resistance. MedComm.

[33] Chen D., Zhang X., Wang X., Ren T., Wang R. (2020). The inhibitory effects of hesperidin and its combination effects with paclitaxel against oral squamous cell car cinoma in nude mice. J. Pract. Stomatol..

[34] Wu D., Li J., Hu X., Ma J., Dong W. (2018). Hesperetin inhibits Eca-109 cell proliferation and invasion by suppressing the PI3K/AKT signaling pathway and synergistically enhances the anti-tumor effect of 5-fluorouracil on esophageal cancer in vitro and in vivo. RSC Adv..

[35] Saleh N., Allam T., Korany R.M.S., Abdelfattah A.M., Omran A.M., Attia Abd Eldaim M., Borai El-Borai N. (2025). Hesperidin exacerbates the therapeutic potency of cisplatin against hepatocytotoxicity of Ehrlich ascites carcinoma in mice. Sci. Rep..

[36] Karabat M.U., Tuncer M.C., Özdemir İ. (2025). In Vitro Evaluation of Cytotoxic and Pro-Apoptotic Effects of Hesperidin Alone and in Combination with Cisplatin on Human Malignant Melanoma Cell Line (A431). Pharmaceuticals.

[37] Çınar R., Yıldızhan K., Altıner H., Yağcı T. (2026). TRPM2 Channel Involvement in the Hesperidin-Mediated Potentiation of Cisplatin’s Antitumor Action in Laryngeal Carcinoma Cells. Int. J. Mol. Sci..

[38] Afşin Y., Özdemir İ., Toprak V., Tuncer M.C., Öztürk Ş. (2025). Combined Hesperidin and Gemcitabine Therapy Modulates Apoptosis and Angiogenesis Pathways in ISHIKAWA Human Endometrial Adenocarcinoma Cells. Medicina.

[39] Özdemir İ., Afşin Y., Tuncer M.C., Öztürk Ş. (2025). Combined Hesperidin and Doxorubicin Treatment Induces Apoptosis and Modulates Inflammatory Cytokines in HeLa Cervical Cancer Cells. Int. J. Mol. Sci..

[40] Sun N., Sun X. (2025). Hesperidin targeting β-catenin inhibits hepatocellular carcinoma stemness and enhances sorafenib sensitivity. Chin. Tradit. Herb. Drugs.

[41] Keyvani-Ghamsari S., Khorsandi K., Rasul A., Zaman M.K. (2021). Current understanding of epigenetics mechanism as a novel target in reducing cancer stem cells resistance. Clin. Epigenet..

[42] Wang S.W., Sheng H., Zheng F., Zhang F. (2021). Hesperetin promotes DOT1L degradation and reduces histone H3K79 methylation to inhibit gastric cancer metastasis. Phytomedicine.

[43] Jurj A., Dragomir M.P., Li Z., Calin G.A. (2026). MicroRNAs in oncology: A translational perspective in the era of AI. Nat. Rev. Clin. Oncol..

[44] Degheidy M.S., Abou-Elalla A.A., Kamel M.M., Abdel-Ghany S., Arneth B., Sabit H. (2025). Regulatory Roles of miR-155-5p, miR-21-5p, miR-93-5p, and miR-140-5p in Breast Cancer Progression. Curr. Issues Mol. Biol..

[45] Magura J., Hassan D., Moodley R., Mackraj I. (2021). Hesperidin-loaded nanoemulsions improve cytotoxicity, induce apoptosis, and downregulate miR-21 and miR-155 expression in MCF-7. J. Microencapsul..

[46] Abdallah R.M., Elkhouly A.M., Soliman R.A., El Mechawy N., El Sebaei A., Motaal A.A., El-Askary H., Youness R.A., Assal R.A. (2022). Hindering the Synchronization Between miR-486-5p and H19 lncRNA by Hesperetin Halts Breast Cancer Aggressiveness Through Tuning ICAM-1. Anticancer Agents Med. Chem..

[47] Saleh N.H., Al-Khafaji A.S.K., Babaei E. (2023). Study of hesperetin effect on modulating transcription levels of MLH1 and MSH2 genes in SKBR3 breast cancer cell line. J. Adv. Pharm. Technol. Res..

[48] Salman A.M., Babaei E., Al-Khafaji A.S.K. (2024). Exploring the modulation of MLH1 and MSH2 gene expression in hesperetin-treated breast cancer cells (BT-474). J. Adv. Pharm. Technol. Res..

[49] Lu Y., Wajapeyee N., Turker M.S., Glazer P.M. (2014). Silencing of the DNA mismatch repair gene MLH1 induced by hypoxic stress in a pathway dependent on the histone demethylase LSD1. Cell Rep..

[50] Vaňková B., Vaněček T., Ptáková N., Hájková V., Dušek M., Michal M., Švajdler P., Daum O., Daumová M., Michal M. (2020). Targeted next generation sequencing of MLH1-deficient, MLH1 promoter hypermethylated, and BRAF/RAS-wild-type colorectal adenocarcinomas is effective in detecting tumors with actionable oncogenic gene fusions. Genes Chromosomes Cancer.

[51] Ghorbani A., Nazari M., Jeddi-Tehrani M., Zand H. (2012). The citrus flavonoid hesperidin induces p53 and inhibits NF-κB activation in order to trigger apoptosis in NALM-6 cells: Involvement of PPARγ-dependent mechanism. Eur. J. Nutr..

[52] Porcuna J., Mínguez-Martínez J., Ricote M. (2021). The PPARα and PPARγ Epigenetic Landscape in Cancer and Immune and Metabolic Disorders. Int. J. Mol. Sci..

[53] Zhang Z., Zhang Y. (2024). Transcriptional regulation of cancer stem cell: Regulatory factors elucidation and cancer treatment strategies. J. Exp. Clin. Cancer Res..

[54] Chatterjee S., Sil P.C. (2019). Targeting the crosstalks of Wnt pathway with Hedgehog and Notch for cancer therapy. Pharmacol. Res..

[55] Borlongan M.C., Wang H. (2023). Profiling and targeting cancer stem cell signaling pathways for cancer therapeutics. Front. Cell Dev. Biol..

[56] Caspa Gokulan R., Devaraj H. (2021). Stem Cell Markers CXCR-4 and CD133 Predict Aggressive Phenotype and Their Double Positivity Indicates Poor Prognosis of Oral Squamous Cell Carcinoma. Cancers.

[57] Choudhury S.D., Ghosh S., Kumar P., Bhardwaj A., Singh K., Singh A., Kumar A., Basu B., Giri R., Choudhury D. (2025). Attenuation of c-Myc expression in breast cancer by hesperidin-mediated stabilization of its promoter proximal G quadruplex region. Int. J. Biol. Macromol..

[58] Xia R., Xu G., Huang Y., Sheng X., Xu X., Lu H. (2018). Hesperidin suppresses the migration and invasion of non-small cell lung cancer cells by inhibiting the SDF-1/CXCR-4 pathway. Life Sci..

[59] Katoh M. (2017). Canonical and non-canonical WNT signaling in cancer stem cells and their niches: Cellular heterogeneity, omics reprogramming, targeted therapy and tumor plasticity (Review). Int. J. Oncol..

[60] Verona F., Di Bella S., Schirano R., Manfredi C., Angeloro F., Bozzari G., Todaro M., Giannini G., Stassi G., Veschi V. (2025). Cancer stem cells and tumor-associated macrophages as mates in tumor progression: Mechanisms of crosstalk and advanced bioinformatic tools to dissect their phenotypes and interaction. Front. Immunol..

[61] Hermawan A., Khumaira A., Ikawati M., Putri H., Jenie R.I., Angraini S.M., Muflikhasari H.A. (2021). Identification of key genes of hesperidin in inhibition of breast cancer stem cells by functional network analysis. Comput. Biol. Chem..

[62] Xie Q., He Z., Tan L., Li M., Zhuang M., Liu C., Chen S., Jin L., Sui Y. (2025). Hesperetin induces apoptosis in lung squamous carcinoma cells via G(2)/M cycle arrest, inhibition of the Notch1 pathway and activation of endoplasmic reticulum stress. Int. J. Mol. Med..

[63] Barat S., Chen X., Cuong Bui K., Bozko P., Götze J., Christgen M., Krech T., Malek N.P., Plentz R.R. (2017). Gamma-Secretase Inhibitor IX (GSI) Impairs Concomitant Activation of Notch and Wnt-Beta-Catenin Pathways in CD44(+) Gastric Cancer Stem Cells. Stem Cells Transl. Med..

[64] Galassi C., Esteller M., Vitale I., Galluzzi L. (2024). Epigenetic control of immunoevasion in cancer stem cells. Trends Cancer.

[65] Li S., Han Z., Zhao N., Zhu B., Zhang Q., Yang X., Sheng D., Hou J., Guo S., Wei L. (2018). Inhibition of DNMT suppresses the stemness of colorectal cancer cells through down-regulating Wnt signaling pathway. Cell Signal.

[66] Huang T., Song X., Xu D., Tiek D., Goenka A., Wu B., Sastry N., Hu B., Cheng S.Y. (2020). Stem cell programs in cancer initiation, progression, and therapy resistance. Theranostics.

[67] Joshi G., Basu A. (2024). Epigenetic control of cell signalling in cancer stem cells. Int. Rev. Cell Mol. Biol..

[68] Galassi C., Manic G., Esteller M., Galluzzi L., Vitale I. (2025). Epigenetic regulation of cancer stemness. Signal Transduct. Target. Ther..

[69] Kyriazi A.A., Papiris E., Kitsos Kalyvianakis K., Sakellaris G., Baritaki S. (2020). Dual Effects of Non-Coding RNAs (ncRNAs) in Cancer Stem Cell Biology. Int. J. Mol. Sci..

[70] Barhela K., Chaudhary A.A., Mishra R., Nupur N., Magani S.K.J., Rudayni H.A., Kumar S., Kumar B. (2025). Interplay between Epigenetic and Transcription Factors Mediating Drug Resistance via Stem Cells in Breast Cancer. ACS Omega.

[71] Tan S., Dai L., Tan P., Liu W., Mu Y., Wang J., Huang X., Hou A. (2020). Hesperidin administration suppresses the proliferation of lung cancer cells by promoting apoptosis via targeting the miR-132/ZEB2 signalling pathway. Int. J. Mol. Med..

[72] Zaghloul R.A., Elsherbiny N.M., Kenawy H.I., El-Karef A., Eissa L.A., El-Shishtawy M.M. (2017). Hepatoprotective effect of hesperidin in hepatocellular carcinoma: Involvement of Wnt signaling pathways. Life Sci..

[73] Roman-Gomez J., Jimenez-Velasco A., Cordeu L., Vilas-Zornoza A., San Jose-Eneriz E., Garate L., Castillejo J.A., Martin V., Prosper F., Heiniger A. (2007). WNT5A, a putative tumour suppressor of lymphoid malignancies, is inactivated by aberrant methylation in acute lymphoblastic leukaemia. Eur. J. Cancer.

[74] Kaviyaprabha R., Miji T.V., Apsara U., Sindhurani S., Sabanayagam R., Muthusami S., Bharathi M. (2025). Inhibitory Effects of Hesperetin and EGCG in the Lung Cancer Progression involves impairment in TOP2A gene expression regulation. Appl. Biochem. Biotechnol..

[75] Roganowicz M., Bär D., Bersaglieri C., Aprigliano R., Santoro R. (2023). BAZ2A-RNA mediated association with TOP2A and KDM1A represses genes implicated in prostate cancer. Life Sci. Alliance.

[76] Kirk J.S., Schaarschuch K., Dalimov Z., Lasorsa E., Ku S., Ramakrishnan S., Hu Q., Azabdaftari G., Wang J., Pili R. (2015). Top2a identifies and provides epigenetic rationale for novel combination therapeutic strategies for aggressive prostate cancer. Oncotarget.

[77] Li L., Du C., Sun N., Xiao X., Li K., Wu H., Gong J. (2026). TOP2A, Stabilized by IGF2BP3 in an m6A-Dependent Manner, Drives Macrophage Recruitment and M2 Polarization in Hepatocellular Carcinoma by YAP1-Mediated CCL2 Activation. Mol. Carcinog..

[78] Chen Q., Hu K., Shi J., Li H., Li W. (2023). Hesperidin inhibits methylation and autophagy in LPS and high glucose-induced human villous trophoblasts. Biochem. Biophys. Res. Commun..

[79] Wu R., Yuan H., Wang Y., Gou X., Hou W., Zhou Z., Wang X., Deng X., Wang C., Wang H. (2025). Norcantharidin inhibits TOP2A expression via H3K27me3 mediated epigenetic regulation to alleviate the progression of hepatocellular carcinoma. Front. Pharmacol..

[80] Li X., Liu Y., Chen W., Fang Y., Xu H., Zhu H.H., Chu M., Li W., Zhuang G., Gao W.Q. (2014). TOP2Ahigh is the phenotype of recurrence and metastasis whereas TOP2Aneg cells represent cancer stem cells in prostate cancer. Oncotarget.

[81] Uusküla-Reimand L., Wilson M.D. (2022). Untangling the roles of TOP2A and TOP2B in transcription and cancer. Sci. Adv..

[82] Güler A.E., Tuncer M.C., Özdemir İ. (2025). Integrated Experimental and Bioinformatic Analysis Reveals Synergistic Apoptotic, Antioxidant, and Immunomodulatory Effects of Hesperidin and Adriamycin in SKOV3 Ovarian Cancer Cells. Biomedicines.

[83] Li J., Xu B., He M., Zong X., Cunningham T., Sha C., Fan Y., Cross R., Hanna J.H., Feng Y. (2021). Control of Foxp3 induction and maintenance by sequential histone acetylation and DNA demethylation. Cell Rep..

[84] Liu S., Zhang C., Zhang K., Gao Y., Wang Z., Li X., Cheng G., Wang S., Xue X., Li W. (2017). FOXP3 inhibits cancer stem cell self-renewal via transcriptional repression of COX2 in colorectal cancer cells. Oncotarget.

[85] Wdowiak K., Walkowiak J., Pietrzak R., Bazan-Woźniak A., Cielecka-Piontek J. (2022). Bioavailability of Hesperidin and Its Aglycone Hesperetin-Compounds Found in Citrus Fruits as a Parameter Conditioning the Pro-Health Potential (Neuroprotective and Antidiabetic Activity)—Mini-Review. Nutrients.

[86] Manach C., Morand C., Gil-Izquierdo A., Bouteloup-Demange C., Rémésy C. (2003). Bioavailability in humans of the flavanones hesperidin and narirutin after the ingestion of two doses of orange juice. Eur. J. Clin. Nutr..

[87] Bussmann A.J.C., Zaninelli T.H., Saraiva-Santos T., Fattori V., Guazelli C.F.S., Bertozzi M.M., Andrade K.C., Ferraz C.R., Camilios-Neto D., Casella A.M.B. (2022). The Flavonoid Hesperidin Methyl Chalcone Targets Cytokines and Oxidative Stress to Reduce Diclofenac-Induced Acute Renal Injury: Contribution of the Nrf2 Redox-Sensitive Pathway. Antioxidants.

[88] Yaghoubi N., Gholamzad A., Naji T., Gholamzad M. (2024). In vitro evaluation of PLGA loaded hesperidin on colorectal cancer cell lines: An insight into nano delivery system. BMC Biotechnol..

[89] Bakhshan M.A., Sheikhzadeh S., Delirezh N. (2025). Enhanced pro-apoptotic and pro-oxidative effects of hesperidin on LNCaP prostate cancer cell line through nano-emulsification. Mol. Biol. Rep..

[90] Deiab N.S., Kodous A.S., Mahfouz M.K., Said A.M., Ghobashy M.M., Abozaid O.A.R. (2024). Smart Hesperidin/Chitosan Nanogel Mitigates Apoptosis and Endoplasmic Reticulum Stress in Fluoride and Aluminum-Induced Testicular Injury. Biol. Trace Elem. Res..

[91] Al-Zuhairy S., Elhabal S.F., Mohamed Elrefai M.F., Hababeh S., Nelson J., Fady M., Elzohairy N.A., Ewedah T.M., Mousa I.S., Hamdan A.M.E. (2025). Polylactic-Co-Glycolic Acid/Alginate/Neem Oil-Reduced Graphene Oxide as a pH-Sensitive Nanocarrier for Hesperidin Drug Delivery: Antimicrobial and Acute Otitis Media Assessments. Pharmaceuticals.

[92] Babaloo H., Barati S., Haghir H., Gholami A.A., Moharreri P., Fallahnezhad S., Asl E.R., Noorzehi G., Tahmasebi F. (2025). The effect of PU/MWCNT nanofiber scaffolds containing hesperidin nanoparticles and mesenchymal stem cells on the microglia and astrocyte phenotype in the spinal cord injury model. Neuroscience.

[93] Huang F.Y., Trumpp A., Stelmach P. (2025). Resolving leukemic stem cell heterogeneity and plasticity with single-cell multiomics. Semin. Hematol..

[94] Fetahu I.S., Esser-Skala W., Dnyansagar R., Sindelar S., Rifatbegovic F., Bileck A., Skos L., Bozsaky E., Lazic D., Shaw L. (2023). Single-cell transcriptomics and epigenomics unravel the role of monocytes in neuroblastoma bone marrow metastasis. Nat. Commun..

[95] Ma H., Zhang V.X., Tsui Y.M., Lee J.M., Lee E., Lu J., Deng H., Zeng F., Ho D.W., Hui C. (2025). Targeting sterol O-acyltransferase 1 rewires fatty acid metabolism and uncovers immune vulnerability in hepatocellular carcinoma. Hepatology.

[96] Fang Y., Gu Y., Xu T., Wang P., Wu X., Shen H., Xu Y., Xu Z., Cao L., Li X. (2025). SENP1 drives glycolysis and cisplatin resistance in gastric cancer via desumoylating ENO1. J. Exp. Clin. Cancer Res..

[97] Rahmani A.H., Babiker A.Y., Anwar S. (2023). Hesperidin, a Bioflavonoid in Cancer Therapy: A Review for a Mechanism of Action through the Modulation of Cell Signaling Pathways. Molecules.

[98] Tabeshpour J., Hosseinzadeh H., Hashemzaei M., Karimi G. (2020). A review of the hepatoprotective effects of hesperidin, a flavanon glycoside in citrus fruits, against natural and chemical toxicities. DARU J. Pharm. Sci..

[99] Zhou Y., Dai Q., Xu Y., Wu S., Cheng M., Zhao B. (2025). PharmaFormer predicts clinical drug responses through transfer learning guided by patient derived organoid. npj Precis. Oncol..

